# Editorial: Women in microbe and virus interactions with plants: 2022/2023

**DOI:** 10.3389/fmicb.2024.1532112

**Published:** 2025-01-09

**Authors:** Sonia Chadha, Esther Menendez, Nuria Montes

**Affiliations:** ^1^Nuclear Agriculture and Biotechnology Division, Bhabha Atomic Research Centre, Mumbai, India; ^2^Homi Bhabha National Institute, Mumbai, India; ^3^Departamento de Microbiología y Genética, Universidad de Salamanca, Salamanca, Spain; ^4^Institute for Agrobiotechnology Research (CIALE), Salamanca, Spain; ^5^Unidad Asociada Grupo de Interacción Planta-Microorganismo, Universidad de Salamanca-IRNASA-CSIC, Salamanca, Spain; ^6^Servicio de Reumatología, Unidad de Metodología, Instituto de Investigación Sanitaria La Princesa (IIS-IP), Madrid, Spain; ^7^Servicio de Reumatología, Hospital Universitario La Princesa, Madrid, Spain; ^8^Plant Physiology, Pharmaceutical and Health Sciences Department, Faculty of Pharmacy, Universidad San Pablo-CEU, CEU-Universities, Boadilla del Monte, Spain

**Keywords:** women scientists, symbiosis, plant promotion, phytoplasmas, plant defense, pathogenicity, plant immune responses, STEM

Frontiers has taken a commendable initiative to celebrate International Women's Day by launching a series of Research Topics designed to highlight and empower women researchers. The Research Topic “*Women in microbe and virus interactions with plants: 2022/2023*” celebrates the women's achievements in this field, providing a platform to showcase and advance their research.

The UNESCO has emphasized the need for gender equality in science as essential for sustainable worldwide development (UNESCO Call to Action, 2024). Gender equality has been discussed for decades, achieving it remains challenging, particularly in STEM (Science, Technology, Engineering, and Mathematics) fields, where women comprise only 30% of the researchers worldwide (UNESCO Institute for Statistics, 2019). Women scientists frequently face obstacles in obtaining authorships, and their contributions are often under evaluated in scientific papers (Chander et al., 2023). This perpetuates a cycle of reduced impact and limited opportunities for women scientists (Sarabi and Smith, 2023). Addressing these challenges is imperative to improve the wellbeing, productivity, and equity of female scientists in STEM (Van den Besselaar and Sandström, 2016).

This Research Topic highlights the impactful contributions of women in the field of plant interactions with microbes and viruses. The Research Topic includes five high-quality research papers that explore the complexities of plant-microbe interactions, including colonization, detection, and disease control, providing critical insights for sustainable agriculture.

Crop production is significantly affected by various pathogens and pests. Addressing these challenges requires a comprehensive understanding of plant-microbe interactions, which encompass competition, commensalism, mutualism, and parasitism. Women scientists have played a pivotal role in advancing our understanding of these processes. The groundbreaking work of Margulis (1967) on symbiosis exemplifies this contribution. Margulis highlighted that cooperation is as important as competition in evolution, an idea that has profoundly influenced our understanding of plant-microbe interactions and has shaped critical areas of plant health and crop productivity.

Plant growth-beneficial bacteria (PGPB) are known to trigger plant metabolism, but their specific interactions and mechanisms remain complex. Galicia-Campos et al. report that *Bacillus* strain H47 significantly increases photosynthetic efficiency, CO_2_ fixation, and transpiration in *Olea europaea* under oxidative stress. It also stimulates the 1-deoxy-D-xylulose 5-phosphate (DOXP) and shikimate pathways and the synthesis of secondary metabolites. Interestingly, despite the significant accumulation of flavonoids in H47-treated olive plants, there is no direct correlation with the expression of flavonoid synthesis genes, suggesting the involvement of a networked pulse activation model similar to that proposed for innate immunity (Kollist et al., 2019; Gutierrez-Albanchez et al., 2020). The study identifies *Bacillus velezensis* H47 as a promising agent for boosting photosynthesis and secondary metabolism in olive trees under stressful environmental conditions.

Phytoplasmas are biotrophic pathogens (Namba, 2019) that reside within the plant's sieve elements (SEs; van Bel and Musetti, 2019; Lewis et al., 2022) and cause various diseases. Due to their minimal genome, they rely on the host's resources. In their study, Musetti et al. showed the interactions between phytoplasmas and the sieve-element endoplasmic reticulum (SE-ER) in the *Arabidopsis thaliana*-“*Candidatus* Phytoplasma asteris” pathosystem (Pagliari et al., 2016, 2021). The authors illustrate that phytoplasma infection leads to substantial morphological changes in the SE-ER. They propose that the phytoplasma effectors induce unfolded protein response (UPR)-related gene expression in companion cells through specialized plasmodesmata. This study provides insight into how phytoplasmas alter host responses to their advantage and enhances our understanding of plant immune responses, paving the way for future research aimed at improving plant defenses against these pathogens.

*Pseudomonas syringae* pv. tomato DC3000 causes bacterial diseases in tomato and *Arabidopsis thaliana*. Its pathogenicity is largely dependent on the bacterial virulence proteins, known as effectors, translocated into the plant cytoplasm through the type III secretion system (T3SS). A novel mechanism uncovered by Marín-Ponce et al. illustrates the disruption of plant defenses by the bacterial type III secreted effectors (T3SEs) HopD1. Previously known to target proteins like NTL9 (Block et al., 2014), HopD1 is now recognized as a disruptor of vesicle trafficking, a critical process for robust immune responses. By highlighting the essential role of AtNHR2B-containing vesicle trafficking in plant defense and their disruption by HopD1, the study advances the understanding of how T3SEs target vesicle trafficking-mediated processes in plants, providing new insights into pathogen strategies and opening new avenues for enhancing plant immune responses.

Cui et al. investigated the relationships among *Diaphorina citri* (*D. citri*), the citrus tristeza virus (CTV; Moreno et al., 2008), and associated endosymbionts, highlighting their roles in citrus Huanglongbing (HLB), the most severe bacterial disease in the citrus industry. CTV causes citrus tristeza disease, a major viral disease in citrus carried by *D. citri*, which is also a vector of citrus HLB pathogen *Candidatus* Liberibacter asiaticus (CLas). The authors reveal that *D. citri* acquires and retains CTV for over 24 days, indicating the virus's persistence and CTV-facilitated transmission of CLas. CTV influences the *D. citri*'s host preferences and alters endosymbiont dynamics, providing critical insights into the interactions that could mitigate CTV transmission and control citrus Huanglongbing disease.

The large-scale use of high-yield varieties, excessive chemical fertilizers, and climate change have transformed the status of minor rice diseases into major threats to rice production. The fungal pathogen *Ustilaginoidea virens* causes false smut in rice, a destructive disease worldwide. The currently employed inoculation methods for investigating pathogen pathogenicity often damage plant tissues, posing a challenge to gaining a deeper understanding of its infection processes. A novel non-destructive panicle sheath instillation technique is presented by Hu et al. to investigate *U. virens* infection stages, providing valuable insights into *U. virens* infection dynamics. The described non-destructive method preserves plant integrity and allows detailed observation of the pathogen's life cycle, marking a significant advancement in the plant-microbe interactions.

In summary, this Research Topic offers fresh insights, recent advancements, and future perspectives in the complex relationships between plants with microbes and viruses, while also addressing current challenges in agriculture and food security. Additionally, by showcasing the latest scientific advances made by women researchers, this Research Topic promotes gender equality and encourages more women to pursue careers in STEM fields.
